# Bovine Coronavirus in diarrheic pre-weaned calves in Egypt: prevalence, risk factors, and the associated biochemical alterations

**DOI:** 10.1007/s11250-025-04331-9

**Published:** 2025-03-12

**Authors:** Magdy M. Elgioushy, Wafaa Hassan, Shimaa M. Abdullah, Hend E. M. Elsheikh, Mahmoud H. Emam

**Affiliations:** 1 Department of Animal Medicine, Division of Internal Medicine, Faculty of Veterinary Medicine, Aswan University, Aswan, 37916 Egypt; 2 Department of Animal Medicine, Division of Internal Medicine, Faculty of Veterinary Medicine, Zagazig University, Zagazig, 44511 Egypt; 3 Department of Animal Medicine, Division of Infectious Diseases, Faculty of Veterinary Medicine, Zagazig University, Zagazig, 44511 Egypt

**Keywords:** Calves, Coronavirus, Diarrhea, Epidemiology, Egypt, Risk factors

## Abstract

Bovine coronavirus (BCoV) is a common viral enteric pathogen responsible for diarrhea in newborn calves. Despite its economic significance, there is limited research on this virus in Egypt. This study aimed to detect the prevalence of BCoV, the associated risk factors, and the biochemical changes during infection. A cross-sectional study included 196 pre-weaned diarrheic calves chosen randomly from 16 farms. Fecal samples were obtained from these diarrheic calves, and a questionnaire was administered to investigate the positivity of BCoV and the potential risk factors. Moreover, blood samples were collected to evaluate the biochemical changes in the infected calves. Logistic regression models were used to assess the strength of the risk factors associated with bovine coronavirus. The prevalence of BCoV among pre-weaned diarrheic calves was 11.22%. The final multivariate analysis revealed that the infection of BCoV was 3.8, 5.96, and 3.2 times higher in males, age ≥ 15 days, and winter season than in female calves, age < 15 days, and other seasons, respectively. The acute phase proteins and the inflammatory biomarkers were changed in infected calves compared to healthy ones. The results indicated that calf age, gender, and exposure to cold temperatures were potential risk factors for BCoV infection. Conversely, no evidence was found to support the hypothesis that BCoV prevalence is linked to locality or ground type. Moreover, the observed biochemical changes in calves with BCoV could assist in the early diagnosis of the infection and provide valuable insights for evaluating prognosis.

## Introduction

Neonatal calf diarrhea (NCD) is the most common cause of calf morbidity and mortality during the first few weeks of life, with significant economic losses (Schild et al. 2020). It results in varying degrees of dehydration, electrolyte imbalances, and metabolic acidosis, adversely affecting the calves' general health and farm profitability (Foster and Smith 2009). Diarrhea in neonatal calves is complex, multifactorial and is influenced by various predisposing non-infectious factors and infectious agents (Cho and Yoon 2014). Different infectious pathogens play a crucial role in the pathogenesis of NCD, such as BCoV, Rotavirus, Cryptosporidium, and E. coli (Brunauer et al. 2021). Also, Identifying the causative agents and risk factors associated with NCD is essential to control diarrheal diseases in calves (Abuelo 2017).

BCoV is a pneumo-enteric virus that affects cattle of all ages, with detrimental effects on the cattle industry (Lotfollahzadeh et al. 2020). The virus causes a high morbidity rate, immune suppression, and decreased growth performance (Alfieri et al. 2018). The virus initially causes yellow to blood-stained mucus-containing diarrhea, progressing into profuse, watery diarrhea (Kapil et al. 1991). Virus shedding occurs in upper respiratory tract secretions and gastrointestinal tract excretion of diseased calves (Hasoksuz et al. 2002). Although the virus is inactivated by heat and common disinfectants, including formalin (Vlasova and Saif 2021), the virus is widely distributed because it can be easily transmitted from carrier cows to their calves through the fecal–oral route (Rocha et al. 2018). Additionally, respiratory transmission of the virus is possible between calves (Hasoksuz et al. 2002). Laboratory serological tests such as ELISA and hemagglutination detect viral antigens (Boileau and Kapil 2010). The haemagglutinin-inhibiting and indirect-ELISA tests that detect antibodies are also diagnostic. Currently, several highly sensitive and specific molecular methods, including polymerase chain reaction (PCR), real-time polymerase chain reaction (RT-PCR), and nested reverse transcription PCR, are widely employed to identify viral RNA (Decaro et al. 2008; Cho et al. 2013; Bok et al. 2015; Gomez et al. 2017).

Several measures are crucial for effectively controlling BCoV infections in diarrheic calves, including isolating infected animals and culling those with persistent diarrhea. The prevention strategy relies on the biosecurity of farms, appropriate colostrum feeding, and good hygienic practices (Hodnik et al. 2020). Several BCoV vaccines have also been developed to prevent and control infection, including vaccinations of cows before calving for passive immunization of calves through colostrum (Tizard 2020). Live attenuated intranasal vaccines were also administered for calves one day or slightly older (Saif 2010). There is a need for better management of neonatal calves, especially the ones more likely to be sick, which would need more attention. Understanding the epidemiology of BCoV and its associated risk factors is critical for developing effective management and treatment strategies. Thus, our primary objective was to detect the prevalence of BCoV among pre-weaned calves in three Egyptian governorates. Our secondary objective was to investigate the risk factors and biochemical changes associated with the infection.

## Material and methods

### Study design and sample size calculation

**Fig. 1:**
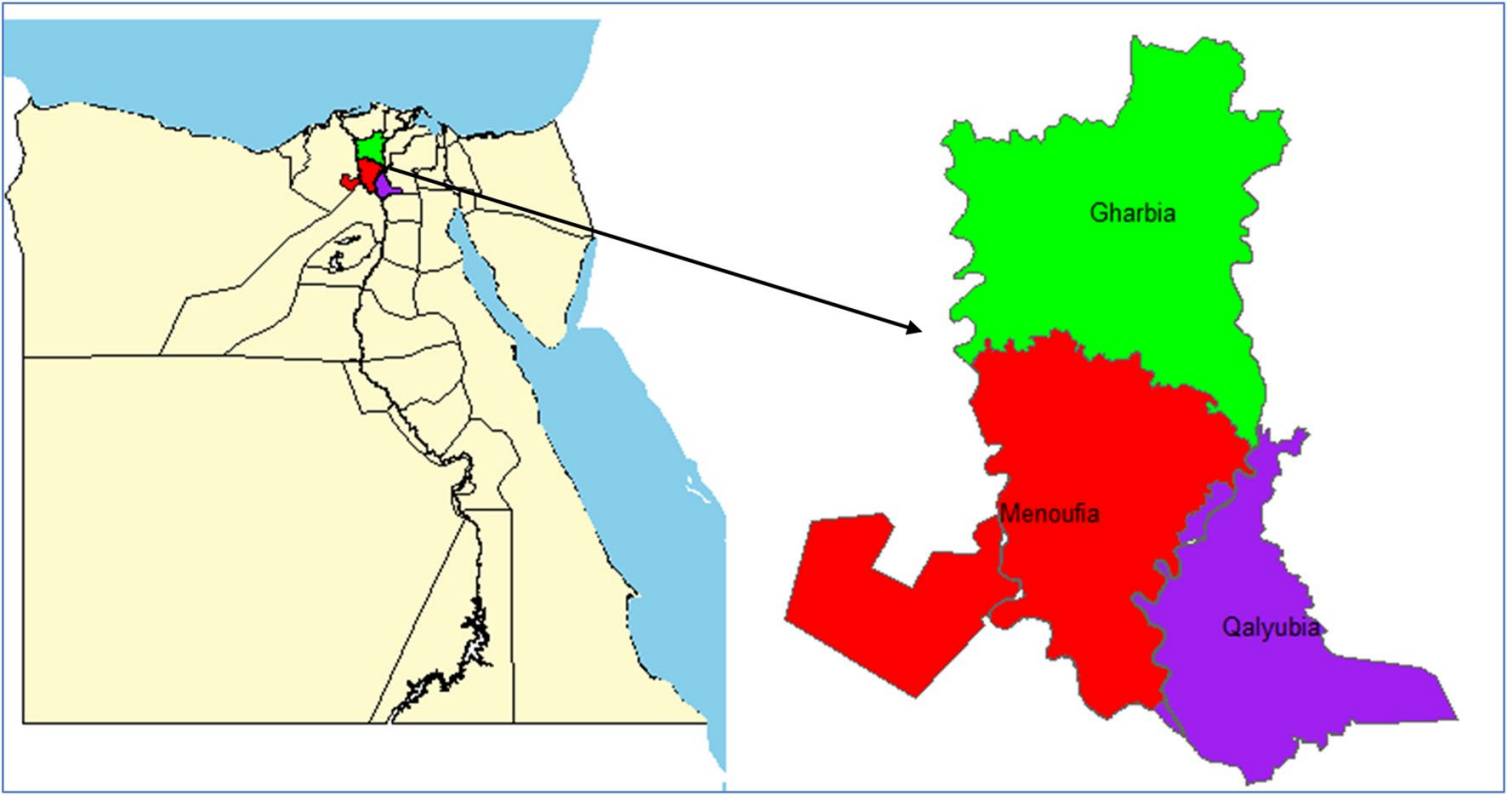
Map of Egypt showing the location of the three investigated governorates; Gharbia, Qalyubia, and Menoufia

The current study was conducted in three governorates in Egypt: Gharbia, Qalyubia, and Menoufia, between March 2022 and October 2024. These governorates are located in the northern part of Egypt and have a hot desert climate according to the Köppen-Geiger climate classification Fig. 1. A total of 196 pre-weaned diarrheic calves were randomly selected from 16 farms located in the investigated governorates. All farms in our study were smallholders and calves were raised in group housing alongside other animals not in individual pens. Also, these farm followed the same traditional rearing, hygienic, and feeding protocol. The number of calves was chosen from each governorate proportionate to the estimated calf population reported by local authorities. Additionally, twenty pre-weaned healthy calves were used as a control group. Animal handling followed the Institutional Animal Care and Use Committee guidelines at Assiut University. The sample size was calculated using Cochran’s formula.

$$\text{n}=\frac{{\text{Z}}^{2} \widehat{p} \left(1-\widehat{p}\right)}{{\text{E}}^{2}}$$

where n = sample size, Z = Z Value is the statistic corresponding to the level of confidence (95% Confidence interval) which is 1.96, p̂ = Population proportion which expected to be 15%, and E = Margin of error (0.05).

## Clinical examination and samples collection

The calves were carefully examined according to the guidelines of Jackson and Cockcroft (Jackson et al. 2002). This clinical examination included rectal temperature measurement, respiratory rate inspection, heart rate auscultation, and dehydration levels evaluation. Diarrhea cases were identified by the presence of liquid or semiliquid stool, a temperature of more than 39.5°C, and diminished appetite. Two blood samples were collected from each calf enrolled in the study. For serum separation, 5 ml of blood was collected into tubes without anticoagulant, and 2 ml of blood was collected in tubes containing sodium citrate for plasma separation. Within two hours of blood collection, centrifugation at 3000 rpm for 20 min was performed to extract serum and separate plasma. The clear serum was extracted from the supernatant using sterile disposable Pasteur pipettes and transferred into 1.5 ml dry sterile Eppendorf tubes. Serum samples were stored at −20°C until biochemical analysis. A questionnaire was designed to identify animal-level risk factors associated with coronavirus diarrhea. Variables, including age (< 15 days and ≥ 15 days), gender, and locality were obtained for all calves included in the study.

### Fecal samples collection and processing

The anal area was wiped clean with a paper towel and then gently probed with a gloved index finger. A sterile tube was used to collect around 30 g of fecal samples. The samples were stored in an ice box before being transferred to the lab for examination. A dilution buffer was added to the feces at a ratio 2:1, then vortexed. The resulting supernatant was carefully drawn off using a pipette and placed in an Eppendorf tube, where it was stored at −80°C until examination.

### Antigen- immunosorbent assay (Antigen -ELI SA)

Bovine coronavirus antigens were identified using Idexx kits (IDEXX laboratories, USA). The tests were performed according to the manufacturer's instructions. Briefly, 50μl of dilution buffer number 8 was added to each well on the plate. The appropriate wells were then filled with 50μl of each positive and negative control sample (duplicate samples were used for the controls). The remaining wells were filled with 50μl of fecal samples. The contents were homogenized using a microplate shaker. The plate was covered with adhesive strips and incubated at 24°C for 30 min. After removing the adhesive strips, liquid was poured from the wells. Each well was washed with 300μl of washing solution, and the solution was drained, repeated 3 times. 100μl of conjugate reagent was applied to each well, followed by incubation at 24°C for 30 min after covering the plate with adhesive strips. After washing the wells 3 times, as previously reported, 100μl of TMB substrate no.9 was added to each well. Away from direct light, the plate was incubated at 24 °C. Finally, 100μl of stop solution was added to each well to stop the reaction. The wells were read by a Microtiter plate reader (Biotech 808) at a wavelength of 450 nm.

### Biochemical analysis

Haptoglobin was measured by the nephelometric method using commercial test kits (Turbox, Orion diagnostica Oy Finland) at a wavelength of 600 nm. The fibrinogen concentration in the citrated plasma was determined using a previously reported technique (Thrall 2005). Serum amyloid A (SAA), total proteins, and albumin levels were measured using commercial test kits through colorimetric methods. The globulin concentration was calculated by subtracting albumin content from the total protein. All biochemical assays were performed precisely following the manufacturer's instructions.

### Statistical analysis

The statistical program SPSS (version 16.0, SPSS Inc., USA) was used to perform all statistical analyses. Logistic regression analysis was conducted to investigate the association between BCoV infection and potential risk factors at the animal level. This analysis involved two stages: univariate logistic regression was used as the first step to identify the individual risk factors for BCoV infection in calves. This statistical method used a dichotomous dependent variable, in this case, a calf's binary categorization as BCoV infected or not, while the independent variables are the indicated risk factors. For independent variables showing a significant relationship (P < 0.1), a multivariate backward stepwise logistic regression analysis was used to determine which variables will be retained in the final models. Results for each variable included the regression coefficient (β), odds ratio (OR) with 95% confidence interval (CI), and standard error. The t-test examined the differences between the calves that had diarrhea and the control group. Mean ± Standard deviation (SD) was used to display the data and P < 0.05 was used to define significance.

## Results

The characteristics and clinical manifestations of diarrheic calves are summarized in Tables 1 and 2. Among the 196 diarrheic calves examined in this study, 22 were infected with BCoV, resulting in a prevalence rate of 11.22%. Calves infected with BCoV exhibited two primary types of diarrhea: watery yellowish diarrhea (68.18%) and pasty yellowish diarrhea (31.82%). Additionally, 22.73% of these calves had severe dehydration, while 77.27% showed mild to moderate dehydration. In contrast, among the 174 diarrheic calves without BCoV, 13.79% showed severe dehydration, while 86.21% had mild to moderate dehydration. Regarding diarrhea types in this group, 38.51% had watery yellowish diarrhea, 17.82% had pasty yellowish diarrhea, and 43.67% had watery greenish diarrhea.

**Table 1:** Diarrheic calves characteristics in the current study including gender, age, weight, degree of dehydration, characters of feces, and bovine corona virus percentage. Among the 196 diarrheic calves examined, 22 were infected with BCoV, with a prevalence rate of (11.22%)

Characteristics	Total (n = 196)	%
Gender:		
Male	118	60.20
Female	78	39.80
Age/day (median)	15	
Weight/Kg (median)	40	
Degree of dehydration:		
Mild	133	67.86
Moderate	47	23.98
Severe	16	8.16
Characters of feces:		
Watery greenish	76	38.76
Watery yellowish	82	41.84
Pasty yellowish	38	19.40
Bovine Corona virus	22	11.22

**Table 2:** Clinical manifestations in calves with bovine coronavirus (BCoV) versus non-bovine coronavirus diarrhea. Calves infected with BCoV had watery yellowish diarrhea (68.18%) and pasty yellowish diarrhea (31.82%). Additionally, 22.73% of these calves had severe dehydration

Variables	Bovine coronavirus positive		Bovine coronavirus negative	
	(*n* = 22)	%	(*n* = 174)	%
Characters of feces:				
Watery greenish	0	0	76	43.67
Watery yellowish	15	68.18	67	38.51
Pasty yellowish	7	31.82	31	17.82
Degree of dehydration:				
Mild	8	36.37	117	67. 24
Moderate	9	40.90	33	18.97
Severe	5	22.73	24	13.79
Fever	9	40.9	46	26.44

### Changes on biochemical parameters

**Fig. 2:**
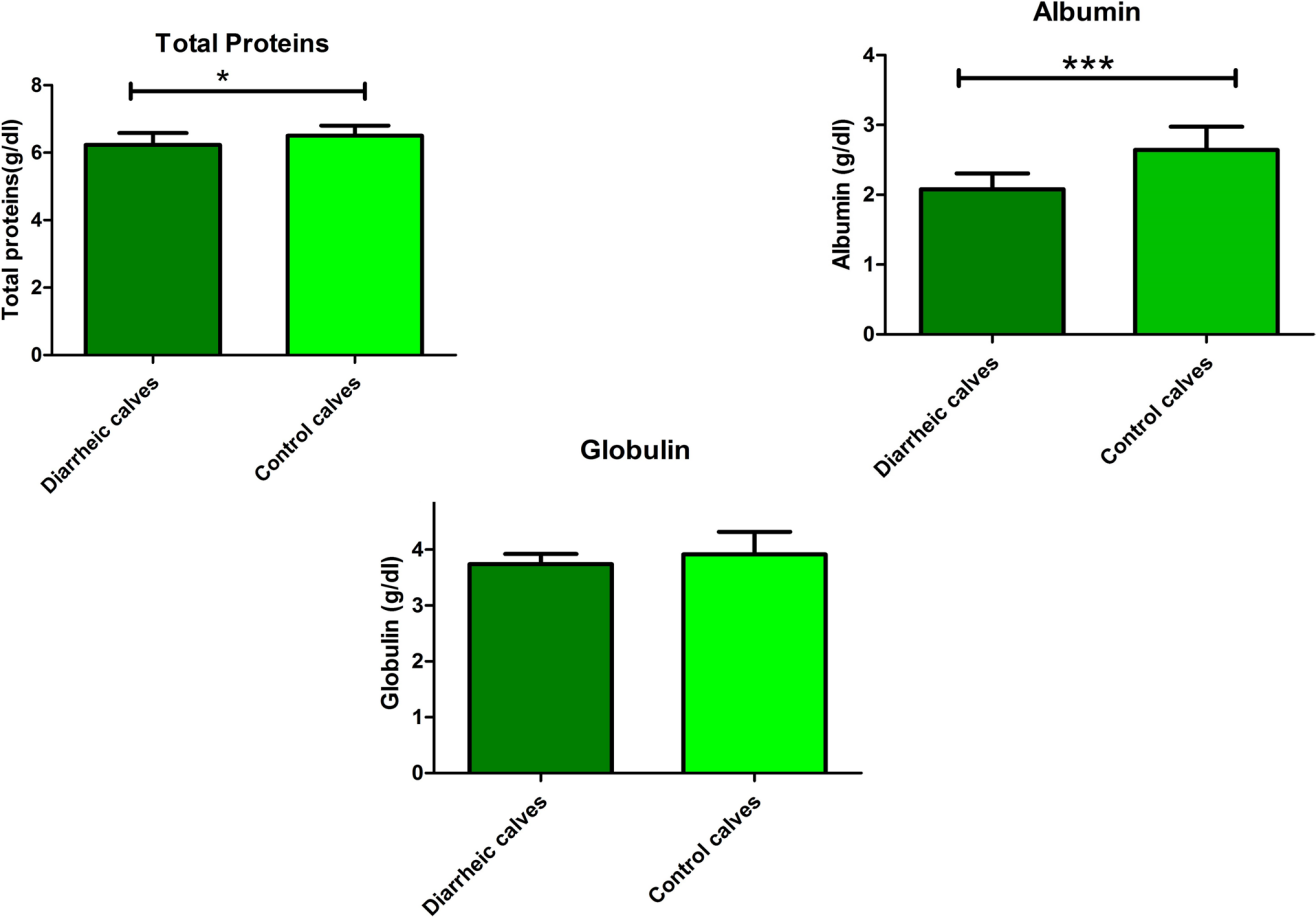
Mean values ± SD of serum total protein (g/dl), serum albumin (g/dl) and serum globulin (g/dl) in control and diarrheic calves. Serum total protein and albumin levels were significantly lower in BCoV-infected calves compared to controls, with *P* values (0.01 and < 0.0001), respectively

**Fig. 3:**
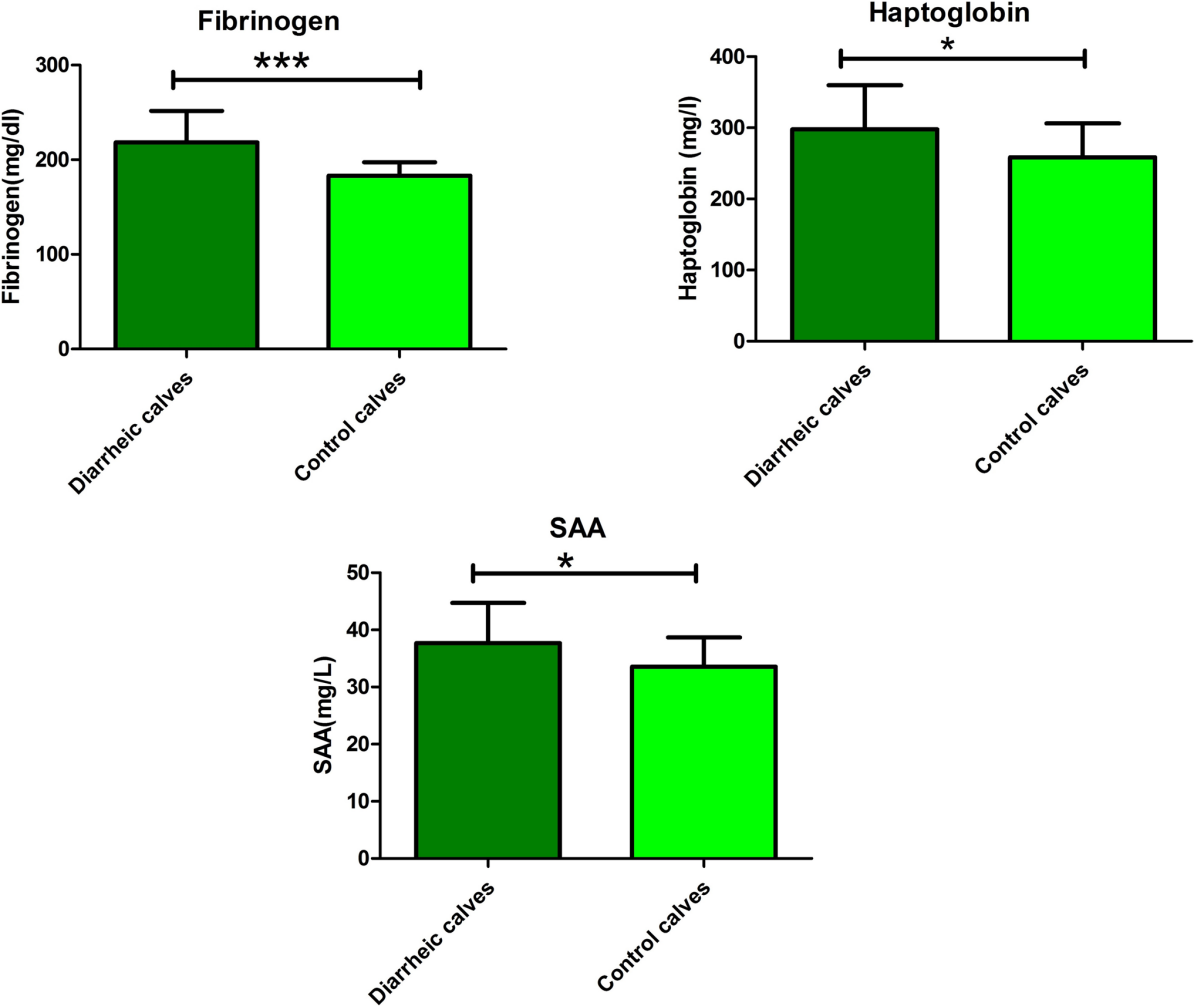
Mean values ± SD of serum haptoglobin (mg/L), SA A (mg/L) and plasma fibrinogen (mg/dl) in control and diarrheic calves. The plasma fibrinogen (*P* < 0.001), haptoglobin (*P* = 0.03), and serum amyloid A (SAA) (*P* = 0.03) showed a significant increase in BCoV-infected calves compared to control calves

Serum total protein and albumin levels were significantly lower in BCoV-infected calves compared to controls, with *P* values (0.01 and < 0.0001), respectively. The mean values ± SD of TP (g/dl) and albumin (g/dl) were (6.2 ± 0.07, 6.5 ± 0.06) and (2.08 ± 0.05, 2.6 ± 0.7) for BCoV and control calves, respectively Fig. 2. The serum globulin levels did not show significant change (*P* = 0.08) in BCoV-infected calves when compared to the control group. However, the plasma fibrinogen (*P* < 0.001), haptoglobin (*P* = 0.03), and serum amyloid A (SAA) (*P* = 0.03) showed a significant increase in BCoV-infected calves compared to control calves. The mean values ± SD of plasma fibrinogen (mg/dl), haptoglobin (mg/l), and SAA (mg/l) were (220 ± 7.8, 180 ± 3.2), (297.9 ± 13.84, 258.5 ± 10.66), and (37.7 ± 1.58, 33.55 ± 1.15) for BCoV and control calves, respectively Fig. 3.

### Descriptive statistics

Table 3 outlined the categorization of diarrheic calves according to their BCoV status. Regarding calves' ages, the prevalence of BCoV was 13.64% and 86.36% in calves under two weeks old and over two weeks old, respectively. The gender-related data showed that 18.20% of female diarrheic calves and 81.80% of male diarrheic calves tested positive for BCoV. Regarding the locality of the farms, the highest prevalence of BCoV was in Gharbia, with 10 cases, followed by 9 cases in Menoufia and only 3 calves in Qalubia. The season-related data showed that winter had the highest BCoV infection rate at 68.18%, while summer had the lowest rate at 4.54%. According to the ground data, 6 calves were positive for BCoV from the total diarrheic calves kept on concrete ground (92/196). In comparison, 16 calves were positive for the infection from the whole diarrheic calves kept on clay ground (104/196).

**Table 3:** Calf diarrhea categorized by BCoV status and the related risk factors associated with the infection. The highest prevalence of BCoV was in calves over 2 weeks age (86.36%), male calves (81.80%), winter season (68.18%), clay ground (72.73%), and Gharbia governorate (45.45%)

Variables	BCoV positive diarrheic calves		BCoV negative diarrheic calves	
	(*n* = 22)	%	(*n* = 174)	%
Age				
< 15 days	3	13.64	76	43.70
≥ 15 days	19	86.36	98	56.30
Gender				
Male	18	81.80	100	57.47
Female	4	18.20	74	42.53
Season				
Summer	1	4.54	17	9.77
Autumn	4	18.18	53	30.46
Winter	15	68.18	75	43.10
Spring	2	9.10	29	16.67
Floor				
Concrete	6	27.27	86	49.43
Clay	16	72.73	88	50.57
Locality				
Gharbia	10	45.45	70	40.23
Menoufia	9	40.91	62	35.63
Qalubia	3	13.64	42	24.14

### Univariate and multivariate regression analysis

The results of the univariate logistic regression model, presented in Table 4, showed a significant association between the sex of calves and infection; the males were more susceptible to infection than females (*P* = 0.021, OR:1.8, 95% CI: 0.71–4.7). Similarly, there was a significant association between BCoV infections and the age of calves. The calves with age ≥ 15 days were more susceptible to the infection than those less than 15 days old age (*P* = 0.013, OR:4.9, 95% CI: 1.4–17.2). Regarding seasonal detection of BCoV, there was a significant association between cold temperature and BCoV infection (*P* = 0.03). The odds ratio for the infection was higher in winter (OR: 2.83, 95% CI: 1.1–7.3) than in the other seasons.

**Table 4:** The Univariate logistic regression models for animal level risk factors associated with bovine coronavirus (BCoV) in diarrheic calves. Significant associations were detected between BCoV infection, and the sex of calves (*P* = 0.021), age (*P* = 0.013) and season (*P* = 0.03)

Variable	β^1^	S.E.^2^	OR^3^	CI 95%^4^		*P-* value^5^
				Lower	Upper	
Gender						
Male vs. female	0.6	0.48	1.8	0.71	4.7	0.021
Age						
≥ 15 days vs < 15 days	1.59	0.64	4.9	1.4	17.2	0.013
Locality						
Gharbia vs others	−0.21	0.45	0.81	0.33	197	0.64
Menoufia vs others	−0.22	0.46	0.80	0.32	1.98	0.63
Qalubia vs others	0.70	0.65	2.02	0.57	7.15	0.28
Season						
Winter vs others	1.04	0.48	2.83	1.1	7.3	0.03
Spring vs others	0.69	0.76	2	0.44	9.02	0.36
Summer vs others	−0.82	1.06	0.44	0.05	3.47	0.43
utumn vs others	0.68	0.58	0.51	0.16	1.57	0.24
Ground						
Clay vs concrete	0.96	0.5	2.6	0.97	6.97	0.051

^1^β: Regression coefficient; ^2^SE: Standard error; ^3^OR: Odds ratio; ^4^CI: confidence interval (95%); ^5^*P*-value < 0.05 were considered significant

The final multivariate models (Table 5) revealed that age, gender, and season were significantly associated with BCoV prevalence (*P* < 0.05). The probability of infection increases with age. Calves (≥ 15 days) were more exposed to infection with BCoV than young animals (< 15 days) by 5.96 times. The odds of disease in males were 3.8 times higher than in females calves. The risk of exposure to infection was higher in winter compared to other seasons. In summary, the odds of infection with BCoV in winter were 3.2 times higher than in different seasons.

**Table 5:** The final multivariate logistic regression models for animal level risk factors associated with bovine corona virus (BCoV) in diarrheic calves. The age, gender, and season were significantly associated with BCoV prevalence (*P* < 0.05)

Variable	β^1^	S.E.^2^	OR^3^	CI 95%^4^		*P-* value^5^
				Lower	Upper	
Gender						
Male vs. female	1.34	0.60	3.8	1.16	12.4	0.027
Age						
≥ 15 days vs < 15 days	1.79	0.66	5.96	1.6	21.8	0.007
Season						
Winter vs others	1.17	0.51	3.2	1.18	8.8	0.022
Ground						
Clay vs concrete	1.03	0.53	2.6	0.99	7.9	0.051
Constant						
	0.29	0.41	1.37			0.478

^1^β: Regression coefficient; ^2^SE: Standard error; ^3^OR: Odds ratio; ^4^CI: confidence interval (95%); ^5^*P*-value < 0.05 were considered significant

## Discussion

The prevalence of BCoV among diarrheic calves was 11.22% in this study, which is different from previous studies in Egypt with various diagnostic tools. The BCoV was detected in diarrhea stool samples from buffalo and cattle calves with a prevalence of 26.8% using real-time polymerase chain reaction (El-Sadek et al. 2019), while a prevalence rate of 3.7% was reported in Dakahlia governorate among diarrheic calves using both Ag-ELISA and RT-PCR (El-Kenawy et al. 2019). On the other hand, the prevalence rate of BCoV among diarrheic calves in Algeria was 20.73%, using indirect antigen-capture ELISA (Ammar et al. 2014). A higher prevalence rate of 33.3% was reported among diarrheic beef calves in Brazil using a semi-nested PCR technique (Lorenzetti et al. 2013). A lower prevalence rate of (2.7%) was reported among diarrheic dairy calves in Iran using antigen capture ELISA (Afshari Safavi et al. 2012). These differences in the prevalence rate of BCoV may be affected by several factors, such as the sample sources, either fecal, serum, or nasal swabs. Serum samples had the highest prevalence of BCoV compared to fecal and nasal samples. Additionally, the prevalence of BCoV varied according to the detection methods, either molecular biology detection, serological testing, or electron microscopy (Geng et al. 2023).

The clinical signs seen in calves infected with BCoV in our investigation, including diarrhea ranging from yellow pasty to yellow watery diarrhea, progressing to profuse watery diarrhea containing mucus and blood, were comparable to the symptoms described in previous research (Saif et al. 1986). Our investigation detected different degrees of dehydration in calves infected with BCoV. Our findings were supported by another study, which reported that insufficient fluid intake plays a significant cause in dehydration, which is detected in BCoV-infected calves (Kapil et al. 1991). The presence of severe, bloody diarrhea can be attributed to the infection of both the small and large intestines by BCoV, which leads to the destruction of villi and results in malabsorption (Torres-Medina et al. 1985). In our study, we reported a significant association between the virus infection rate and calf gender. Previous research supported these findings; the authors reported a higher infection rate of BCoV in males compared to females (Ammar et al. 2014). Furthermore, the authors attributed this increase to the larger size of male calves at birth, which can lead to dystocia and decreased colostrum absorption. In contrast, previous reports by Afshari Safavi et al. (2012) and Gomez et al. (2017) reported no significant association between BCoV infection rates and the gender of examined calves.

The significant association between colder temperatures and BCoV infection reported in this study aligns with previous investigations, which found that BCoV outbreaks were more common in the winter than the summer (Hasoksuz et al. 2002; Takiuchi et al. 2006; Amer 2018). The ability of BCoV to survive better in environments with low temperatures and low UV levels explains the high prevalence in winter (Williams and Barker 2008). On the other hand, a previous study did not detect any significant association between BCoV prevalence and season (Moosakhani et al. 2012). However, the summer season was the most prevalent time of BCoV infection in calves, according to Boileau and Kapil (2010) and Abbas et al. (2024).

The increased risk of BCoV infection in calves aged from 15 up to 30 days old compared to those aged less than 15 days old reported in this study is consistent with a previous report; the authors concluded that calves with more than 2 weeks age old had the highest percentage of positive samples 29% (Stipp et al. 2009). The variation in the infection rate may be attributed to the immune status of calves, which is higher in the first week of life due to passive immunity provided by colostrum antibodies in the lumen of the intestine of the newborn (Ammar et al. 2014). While colostrum antibodies in serum do not directly protect, they contribute to mucosal immunity through re-secretion into the gut lumen. The amount of immunoglobulin in the lumen of the intestine determines the protection against clinical diseases (Constable et al. 2016). On the contrary, a recent study did not detect any significant association between calves' age and BCoV infection rate (Lotfy et al. 2020). Meanwhile, previous work reported a higher prevalence of Coronavirus infection in dairy during their first 10 days of life (Cruvinel et al. 2020). The high prevalence in this age (< 10 days) may be attributed to the impairment of the immune status of these calves, which is simultaneously affected by the mixed infection of viruses, bacteria, and protozoa.

Although the current study did not find any significant association between ground type and BCoV infection, calves reared on clay ground had a numerically higher infection rate compared to those reared on concrete ground, with rates of 15.4% and 6.5%, respectively. Previous studies reported that BCoV may remain infectious for up to 3 days in the presence of organic materials (Clark et al. 1998). Furthermore, BCoV can be concentrated on the surface of the clay, resulting in potential exposure of cattle to infectious doses of BCoV through contact with the soils (Geng et al. 2023). Similarly, we did not report any significant association between locality and BCoV infection rate; these findings are in line with that reported in cattle and buffaloes by (Ferrara et al. 2023), who reported a lack of significant differences in BCoV infection rates in different provinces in southern Italy. The lack of differences may be attributed to the same environmental conditions of the various regions included in the study.

Analysis of biochemical markers is crucial for assessing bovine health. Furthermore, accurately interpreting these biomarkers changes can provide more information about disease status and prognosis (Gånheim et al. 2007; Eckersall and Bell 2010). In the current study, we detected significant changes in the level of inflammatory biomarkers, which reflect the immune system's rapid response after the infection. Our findings were supported by previous reports (Pourjafar et al. 2011; Aydin et al. 2022), which reported that SAA and haptoglobin levels were higher in calves infected with BCoV than in healthy calves. These studies suggested that the increased SAA is primarily due to lipopolysaccharide release during diarrhea, stimulating inflammatory biomarker secretion. Conversely, Chae et al. (2019) reported no changes in the levels of SAA in calves suffering from bovine coronavirus diarrhea. They concluded that haptoglobin levels were a more reliable indicator of neonatal calf health.

Calves should be reared in separate pens until three weeks of age, limiting the direct contact between animals to minimize transmission of viral infections and reduce neonatal calves diarrhea (Curtis et al. 2016; Bertoni et al. 2021). The investigated calves in the current study were from smallholder farms in the northern governorates. Furthermore, these calves raised in grouped housing with other animals, not in individual pens. These managemental protocols may contribute to the positive status of BCoV infection and impede effective prevention. Therefore, our findings should be treated with caution. Further investigation with a larger sample of calves from different farms is recommended.

## Conclusion

We concluded that calf age, gender, and the winter season are potential risk factors contributing to the prevalence of BCoV. Additionally, inflammatory alterations observed during infection may serve as biomarkers for BCoV epidemiology. Our findings highlight the importance of enhancing calf immunity, controlling infections during the winter, and providing particular attention to male calves and calves more than 2 weeks age to reduce BCoV infection rates in neonatal calves. These insights can inform future management and control strategies, ultimately improving farm profitability by reducing morbidity and mortality rates. Future investigations with a larger sample of calves from different farms are recommended to explore genetic factors influencing susceptibility to BCoV and the role of colostrum quality in early immunity for reducing BCoV infection.

## Data Availability

All data findings during our investigation are available within the manuscript.
